# Colonic Derotation Revisited: The "Deloyers Procedure" for Long-Segment Hirschsprung Disease

**DOI:** 10.7759/cureus.75539

**Published:** 2024-12-11

**Authors:** Ismael Elhalaby, Irene Isabel P Lim, Elizaveta Bokova, Ifeanyi K Egbuchulem, Rebecca M Rentea

**Affiliations:** 1 Comprehensive Colorectal Center, Department of Surgery, Children's Mercy Kansas City, Kansas City, USA; 2 Department of Surgery, Faculty of Medicine, Tanta University, Tanta, EGY; 3 Department of Surgery, University of Missouri-Kansas City, Kansas City, USA; 4 Division of Pediatric Surgery, Department of Surgery, University College Hospital, Ibadan, NGA

**Keywords:** colonic derotation, deloyers procedure, long-segment hirschsprung disease, operative technique, pull-through, turnbull procedure

## Abstract

Long-segment Hirschsprung disease (HSCR) presents significant challenges in surgical management, often requiring extensive bowel mobilization and creative techniques to achieve tension-free anastomosis. Colonic derotation offers a viable solution for preserving bowel length and maintaining the ileocecal valve, which is crucial for postoperative bowel function. The procedure involves extensive colonic mobilization and strategic vascular divisions of the right and middle colic vessels while preserving the ileocolic and marginal arteries, followed by a 180° counterclockwise rotation of the colon around the ileocolic vascular axis. Critical aspects include ensuring proper bowel orientation post-derotation, the careful assessment of potential compression points, and the preservation of the appendix for potential future antegrade enema conduit creation. The procedure concludes with a transanal pull-through and coloanal anastomosis, achieving optimal bowel positioning without undue tension or laxity. This technique provides a valuable option in the surgical armamentarium for managing complex cases of long-segment Hirschsprung disease.

## Introduction

Hirschsprung disease (HSCR) is a rare congenital disorder of the enteric nervous system, occurring in approximately one in 5,000 live births [1]. In about 10% of children with HSCR, the affected segment extends proximal to the splenic flexure, presenting significant surgical challenges. In such cases, ensuring a well-vascularized, tension-free coloanal anastomosis following an extended colectomy can be difficult due to the limited length of the remaining colon [2].

First reported in 1958, colonic derotation, also known as the Deloyers procedure and described in detail in the adult literature [3], has been widely utilized to preserve colon length after extensive colonic resection for conditions such as colon cancer and diverticulosis [4-6]. This technique has also been adopted in pediatric colorectal surgery to manage long-segment HSCR and idiopathic constipation requiring extensive colon resections [7-10]. The procedure involves the complete mobilization and counterclockwise rotation of the right colon, allowing the remaining colonic segment to be brought into the pelvis with an isoperistaltic anastomosis. Pediatric surgeons often lack experience with this technique, and its consideration in preoperative planning is limited, partly due to the scarcity of detailed operative descriptions in pediatric surgical literature. This technical report revisits the application of colonic derotation in children with long-segment HSCR and highlights key intraoperative technical milestones.

## Technical report

Description of operative technique

Colonic Mobilization

**Figure 1:**
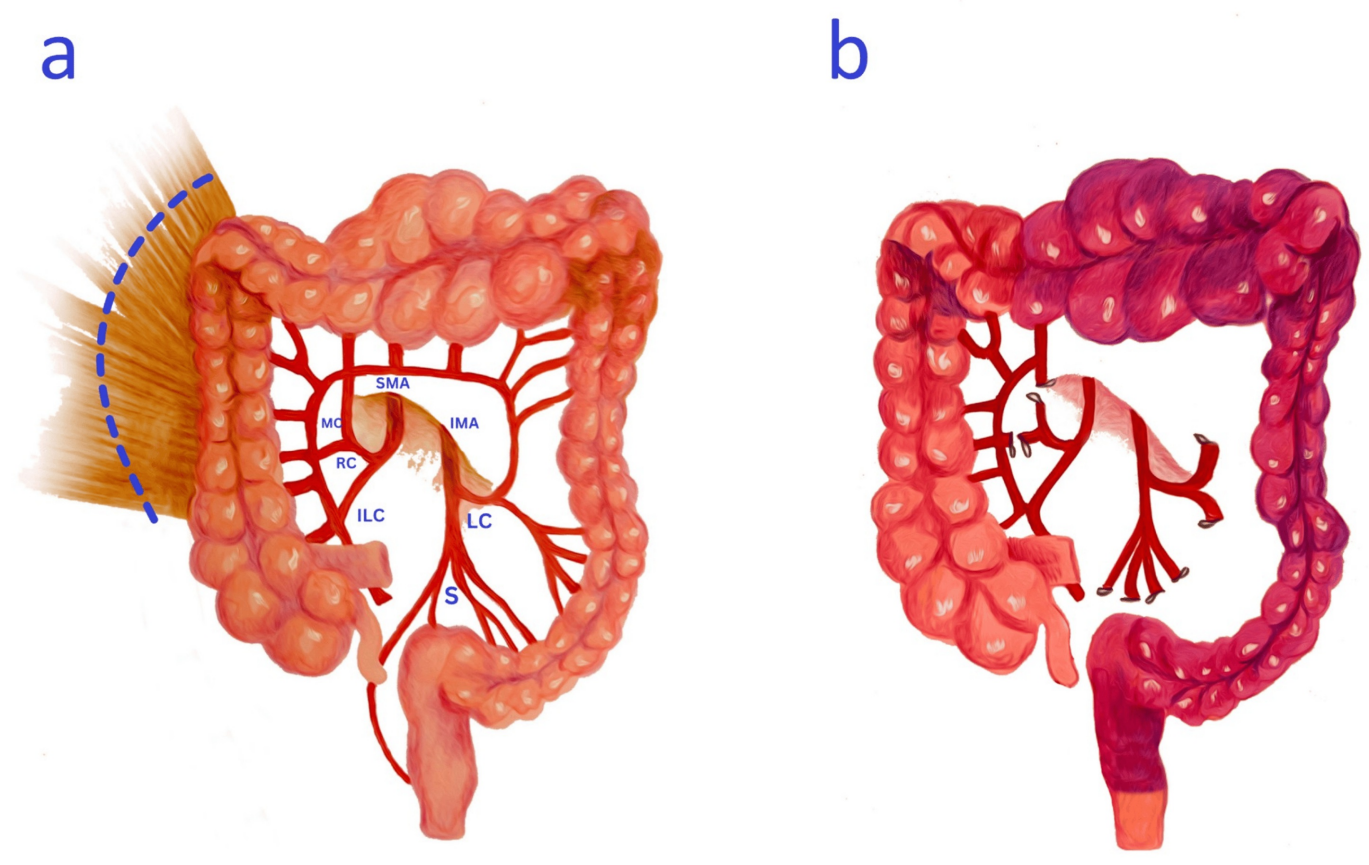
Colonic mobilization and vascular divisions (A) The transition zone is demonstrated in the transverse colon with a normal ganglionated colon at the proximal transverse colon. The right colon and hepatic flexure are mobilized from their lateral attachments. Key vascular structures are identified: SMA, superior mesenteric artery; MC, middle colic artery; ILC, ileocolic artery; IMA, inferior mesenteric artery; LC, left colic artery; RC, right colic artery; and S, sigmoidal branches. (B) The right and middle colic arteries are divided, and thus, the sole blood supply of the right colon is from the ileocolic artery and the intact marginal artery. The anal canal is supplied by the intact hemorrhoidal vessels. Image credits: Ismael Elhalaby, Irene Isabel P. Lim, Elizaveta Bokova, Ifeanyi K. Egbuchulem, and Rebecca M. Rentea

The initial step of the procedure involves the mobilization of both the aganglionic and ganglionated segments of the colon. Mobilization starts at the sigmoid colon by creating a mesenteric window, allowing the division of the mesentery and the circumferential dissection of the colon toward the pelvic brim. Dissection then continues through the mesorectal plane and down to the pelvic floor while maintaining close proximity to the bowel wall to avoid injury to the ureters, bladder, and nerves. Attention is then directed proximally, taking care to mobilize the descending colon from the peritoneal attachments to the splenic flexure. The mesentery is divided to the level of the middle colic artery. The gastrocolic ligament is divided, followed by the right phrenicocolic ligament to fully mobilize the hepatic flexure. The ganglionated proximal right colon is mobilized along the lateral aspect by incising the attachments from the cecum to the hepatic flexure along the white line of Toldt, taking care to protect the duodenum and right ureter (Figure 1A). Ileostomies, if created prior, should be mobilized to allow for appropriate bowel derotation.

Vascular Divisions

The careful planning of the vascular divisions follows complete colon mobilization. The right colon receives blood supply from the ileocolic, right, and middle colic arteries, with the marginal artery providing a source of collateral circulation between their branches. The goal of the vascular divisions is to further mobilize the colonic segment that will be pulled through to achieve a tension-free anastomosis while ensuring adequate blood supply. After assessing the integrity of the marginal artery, the right branch of the middle colic artery and the right colic artery, along with their corresponding veins, are divided. The colon is then divided at the site of confirmed ganglion cells as per prior preoperative mapping, and its blood supply is now left reliant on the ileocolic artery and the marginal artery (Figure 1B). Further confirmation of adequate blood supply can be performed using indocyanine green (ICG) fluorescence, if available.

Colonic Derotation

**Figure 2:**
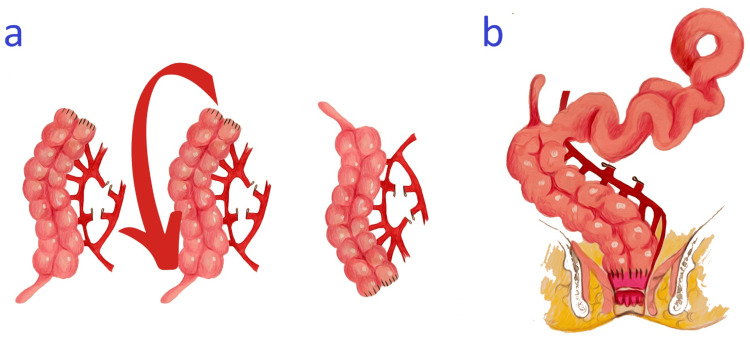
Colonic derotation and establishing bowel orientation (A) Following transection at the site of confirmed ganglion cells, the colon is rotated 180° anticlockwise around the intact ileocolic vascular axis. (B) The derotated colon is brought along the right side of the abdomen and secured to the posterolateral abdominal wall. The small bowel is placed on the left side of the abdomen. Coloanal anastomosis is performed proximal to the dentate line. Image credits: Ismael Elhalaby, Irene Isabel P. Lim, Elizaveta Bokova, Ifeanyi K. Egbuchulem, and Rebecca M. Rentea

After colonic mobilization and vascular divisions, the remaining colon is rotated 180° anticlockwise based on the intact ileocolic vascular axis (Figure 2A). At the completion of this rotation, the cecum is positioned in the subhepatic space. The anterior aspect of the right mesocolon would lie in the right paracolic gutter, and the posterior aspect of the mesocolon becomes anterior. Likewise, the anterior surface of the cecum would now be facing the retroperitoneum.

Establishing Bowel Orientation

Following the derotation of the colon, bowel orientation is carefully inspected. The derotated colon should now lie on the right side of the abdominal cavity, while the entirety of the small bowel is oriented to the patient's left side. A thorough inspection from the duodenum distally is necessary to check for potential bowel compression or vascular twisting brought by the derotation (Figure 2B). The pulled-through right colon would be eventually fixed to the right paracolic gutter to guard against any vascular twisting or small bowel internal herniation. The procedure concludes with the transanal dissection within the Swenson (full-thickness) plane. An isoperistaltic tension-free anastomosis is performed between the pulled-through segment and the anal canal.

## Discussion

Colonic derotation is a technically demanding procedure that requires meticulous planning and expertise. Its utilization in the pediatric population is rare, and thus, highlighting its technical nuances and potential challenges is imperative for optimal outcomes.

**Figure 3:**
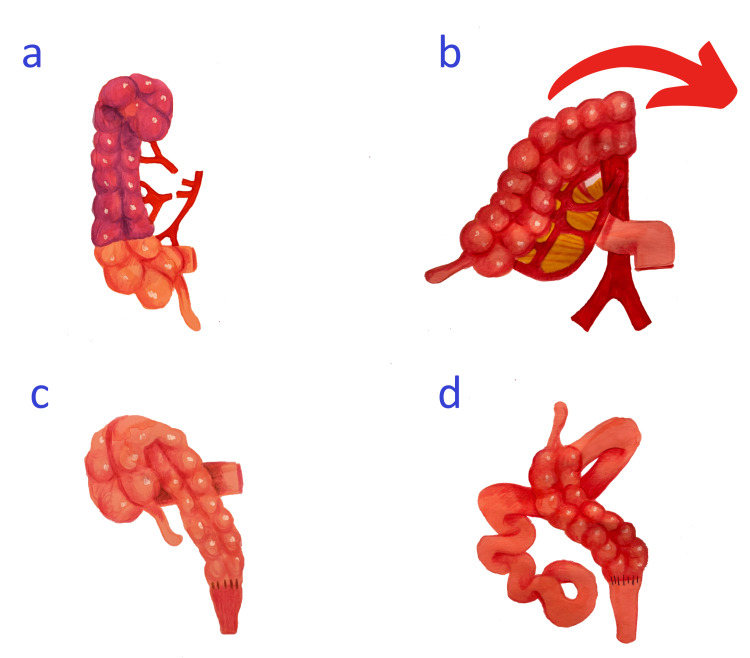
Potential pitfalls in colonic derotation (A) Vascular compromise: ligating the right colic and middle colic artery in the absence of adequate marginal artery results in the ischemia of the distal right colon. (B) Duodenal compression: pulling the mobilized colon to the left side results in the compression of the third portion of the duodenum by the middle colic artery. (C) Ileal compression: attempting a clockwise rotation of the mobilized colon results in the compression of the terminal ileum. (D) Internal herniation: failure to establish proper bowel orientation can lead to the internal herniation of the small bowel posterior and lateral to the pulled-through colon, resulting in obstruction or ischemia. The small bowel should be positioned on the patient's left side, with none lateral to the plane of the right colon. Image credits: Ismael Elhalaby, Irene Isabel P. Lim, Elizaveta Bokova, Ifeanyi K. Egbuchulem, and Rebecca M. Rentea

Ensuring adequate blood supply is of utmost importance. The torsion of vessels or improper vasculature control can result in subsequent hypoperfusion, necrosis of the remaining colon, and anastomotic leak. Although this was not reported in the previous series, it is still pertinent to cautiously assess the integrity of the marginal artery since variabilities in the marginal arcade anatomy have been studied [5,6,11]. In one study, the marginal artery establishing anastomosis between the ileocolic and right colic arteries was adequate in only 30% of cases [12]. Accordingly, if this vascular cross-communication is absent, the ligation of the middle and right colic arteries can jeopardize the blood supply to the distal right colon risking subsequent ischemia (Figure 3A). Such concerns have led adult colorectal surgeons to utilize adjuncts such as indocyanine green fluorescence to assure adequate vascularity following colonic derotation with satisfactory results [13,14]. While ICG utilization has been studied in various pediatric colorectal procedures, its application in HSCR patients undergoing this specific intervention remains largely unexplored [15]. Preliminary observations suggest promising results, warranting further investigation [8].

Avoiding the inadvertent compression of small bowel segments is another critical consideration. The pulled-through colon must be brought down on the right side of the pelvis since bringing it on the left side can cause the compression of the third portion of the duodenum by the middle colic or the ileocolic arteries, resulting in potential obstruction (Figure 3B). Similarly, attempting a clockwise rotation of the distal colon to bring it down in the pelvis carries a significant risk of terminal ileum compression by the pulled-through colon leading to bowel obstruction symptoms (Figure 3C). It is also critical to check the final orientation of the pulled-through colon, making sure that it lays against the right paracolic gutter without undue tension or laxity. Failure to do so may result in the small bowel herniating behind the right colon with subsequent obstruction and/or ischemia (Figure 3D).

When faced with cases of long-segment aganglionosis, surgeons may consider other maneuvers such as transmesenteric lowering or total colectomy, but these have specific implications in HSCR. The transmesenteric lowering, which consists of passing the proximal colon through an ileal avascular mesenteric window, was first described by Rombeau et al. in 1978 [16]. While it can serve as an alternative to the Deloyers procedure, this technique requires the presence of the entire transverse colon, which may not be feasible in long-segment HSCR. It is associated with a potential risk of internal herniation through the mesenteric window and a risk of small bowel loop obstruction. Although a recent study demonstrated the feasibility and favorable outcomes of this technique in select cases of long-segment Hirschsprung disease, there remains a paucity of literature on its broader application in the pediatric population [9]. The resection of the entire colon with an ileorectal anastomosis may pose problems for pediatric patients owing to the higher frequency of bowel movements and decreased stool consistency [17]. This can result in severe perineal rash and difficulty with continence (even with an intact anal canal and normal sphincters), as well as a higher risk for growth failure [18]. Accordingly, the preservation of the ileocecal junction, which acts as a sphincter, prevents a rapid inflow of ileal content in the cecum and results in better functional outcomes and quality of life compared to ileorectal anastomosis [6,19].

The decision to perform appendectomy during colonic derotation is critical in pediatric HSCR management. While Deloyers' original description did not address this step, many adult surgeons routinely perform appendectomy to mitigate potential diagnostic and management challenges of future appendicitis in its new pelvic location post-pull-through. In children with HSCR, the preservation of the appendix is essential for potential antegrade continence enema conduit creation. Furthermore, appendectomy poses a theoretical risk of appendiceal stump blowout in the event of postoperative obstructive symptoms.

The expanding role of minimally invasive pediatric colorectal surgery brings to question laparoscopic approaches for colonic derotation in long-segment HSCR. However, several factors unique to the pediatric population present significant challenges: the limited intra-abdominal working space, potential adhesions from prior interventions, and the extensive mobilization required for these cases. Despite these challenges, recent studies have demonstrated the feasibility and safety of laparoscopic colonic derotation for long-segment HSCR when performed by surgeons with appropriate expertise and setup [8,20]. Further research is essential to validate these findings and should focus on long-term outcomes and optimal patient selection criteria for laparoscopic colonic derotation in long-segment Hirschsprung disease.

Finally, while this report focuses on the technical aspects of the Deloyers procedure, it is prudent to highlight potential postoperative complications that may occur following any pull-through procedure for Hirschsprung disease. These can include an anastomotic leak, an anastomotic stricture, a pull-through twist, a retained Yancey-Soave cuff, or a Duhamel spur [21]. Given that complications can occur with any pull-through technique and studies show comparable outcomes, surgeons should perform the procedure that they are most experienced with while remaining vigilant about potential complications and implementing effective prevention and management strategies [22,23].

## Conclusions

Colonic derotation offers significant advantages in managing long-segment Hirschsprung disease over alternative techniques such as transmesenteric lowering or total colectomy by preserving bowel length and the ileocecal valve. While technically demanding, this procedure can yield optimal functional outcomes when executed meticulously. Key considerations include the careful assessment of vascular integrity particularly the marginal artery, precise bowel derotation technique, and ensuring proper orientation of the derotated colon and bowel to prevent potential complications such as internal herniation or the compression of adjacent structures. The preservation of the appendix is of particular importance, as it may serve as a conduit for future antegrade continence enema procedures. Future refinements may incorporate minimally invasive approaches, although significant challenges persist due to the limited working space and potential adhesions in these complex cases.
